# Beyond Glycemic Control: Vascular Biologic Effects of Antidiabetic Drugs and Their Cardiovascular Promise

**DOI:** 10.31083/RCM46451

**Published:** 2025-10-28

**Authors:** Kelsey C. Muir, Christopher Stone, Frank W. Sellke

**Affiliations:** ^1^Department of Surgery, Division of Cardiothoracic Surgery, Warren Alpert Medical School, Brown University, Providence, RI 02912, USA; ^2^Cardiovascular Research Center, Rhode Island Hospital, Providence, RI 02903, USA

Ischemic heart disease (IHD) remains a leading cause of morbidity and mortality worldwide. Despite major advances in revascularization and pharmacotherapy, numerous patients are left with residual disease or symptoms [1, 2]. Coronary angiogenesis and microvascular function are critical determinants of myocardial perfusion yet they remain insufficiently investigated. In recent years, antidiabetic drugs, including sodium-glucose cotransporter (SGLT) inhibitors, dipeptidyl peptidase-4 (DPP-4) inhibitors, and glucagon-like peptide-1 (GLP-1) receptor agonists, have demonstrated cardiovascular benefits that extend beyond glycemic control. While their benefit in heart failure and cardiovascular outcomes are well established [3, 4, 5, 6, 7], their direct vascular and angiogenic effects are less defined. Preclinical and translational evidence suggests these agents influence endothelial function, collateral vessel formation, and maladaptive remodeling. The evidence underscores the need for future clinical investigations to clarify their vascular effects and define the ideal patient that will most benefit from them.

The myocardium adapts to ischemia through angiogenesis, collateral vessel formation, and microvascular vasoreactivity, all of which determine perfusion. Therapeutic angiogenesis (TA) has emerged as a potential solution; however, it has not been adopted clinically, due to issues with patient selection, trial design, and reproducibility of biologic effects [8, 9, 10, 11]. Angiogenesis is manifested by endothelial proliferation, migration, and survival, stabilization and maturation of nascent vessels, and complex interactions between growth factors, extracellular matrix, and inflammatory mediators [12]. On the other hand, maladaptive angiogenesis, characterized by disorganized or inadequate vessel formation, fails to restore perfusion or may worsen myocardial injury. More sophisticated strategies, including combined protein and stem cell delivery or engineered extracellular vesicles, are under investigation [9]. Collectively, these interventions emphasize that restoring coronary microvascular environment requires an integrated and multifaceted approach rather than delivery of a single factor. Against this backdrop, the vascular effects of novel antidiabetic drugs warrant close consideration.

Large cardiovascular outcome trials (CVOTs), such as SELECT or EMPA-REG OUTCOME [4, 7], demonstrated reduced major cardiovascular events in patients with diabetes or obesity. Growing evidence suggests these benefits extend beyond their glycemic control and involve direct effects on vascular biology. For example, in a swine model of chronic myocardial ischemia, the SGLT2 inhibitor canagliflozin improved perfusion, attenuated fibrosis, and enhanced ventricular function, even without overt angiogenesis [13]. Complementary work in rodent models showed canagliflozin improved absolute myocardial blood flow through enhanced microvascular vasodilation, highlighting its ability to restore microvascular reactivity independent of angiogenesis [14]. In swine with diet-induced metabolic syndrome, canagliflozin demonstrated increased capillary density in ischemic myocardium, demonstrating angiogenic potential under metabolic stress [15]. Importantly, human studies have shown that SGLT2 inhibition enhances coronary microcirculation, endothelial homeostasis, and contractile performance [16, 17]. Similarly, GLP-1 receptor agonists and DPP-4 inhibitors appear to modulate vascular biology. In our swine model of IHD, semaglutide improved perfusion and ventricular function, with reduced fibrosis and enhanced endothelial signaling [18]. These findings highlight the multifaceted mechanisms by which GLP-1 agonists may protect the ischemic myocardium. Linagliptin, a DPP-4 inhibitor, reduced fibrosis and apoptosis while improving overall cardiac performance, illustrating its favorable vascular effects [19]. Human data remains limited but shows promise. A pilot trial of liraglutide in 24 patients with type 2 diabetes, although underpowered, suggested increased coronary microvascular function [20]. Another recent study showed dulaglutide enhanced endothelial function and indices of arterial stiffness [21]. Collectively, these findings suggest SGLT2 inhibitors, GLP-1 receptor agonists, and DPP-4 inhibitors exert cardiovascular benefits in part by modulating microvascular reactivity, endothelial homeostasis, and angiogenesis. Their utility appears most relevant in patients with diffuse coronary disease or microvascular angina, where impaired angiogenesis and vascular reactivity sustain ischemic burden. Additionally, patients with diabetes or metabolic syndrome, who often exhibit marked impairment in endothelial function and vascular reactivity, may especially benefit from these therapies [22].

Combination therapy with traditional antidiabetic medications, such as metformin, may augment these effects. In our swine model of IHD, metformin alone improved cardiac function by improving perfusion and reducing apoptosis, independent of glycemic control [23]. These findings align with historical evidence linking metformin to improved cardiovascular outcomes, and more recent studies demonstrating enhanced microvascular and endothelial function [24, 25, 26, 27, 28]. Notably, most participants in CVOTs of SGLT2 inhibitors and GLP-1 agonists were receiving metformin (67–82%), suggesting it may contribute to the cardiovascular benefits of these newer drugs [29]. Therefore, delineating how these mechanistic pathways interact across various drug classes will be critical to guide their role in IHD.

The vascular and angiogenic effects of these drugs raise an important question: in which patients will cardiovascular benefits be most meaningful? To date, CVOTs have focused on heart failure and atherosclerotic events, but patients with diffuse coronary disease or microvascular angina, conditions where ischemia persists without discrete lesions amenable to revascularization, may represent a particularly important target population. Future trials should move beyond broad outcomes to incorporate mechanistic endpoints, including myocardial perfusion imaging, endothelial function testing, and circulating angiogenic biomarkers. Crucial endpoints can be assessed using invasive techniques, such as quantitative coronary angiography with infusion of vasoactive agents to directly measure endothelial function, intravascular ultrasound to characterize lumen morphology following vasoactive challenges, or fractional flow reserve [30, 31]. Additional secondary endpoints may also be captured through non-invasive techniques, including positron emission tomography and single-photon emission computed tomography (PET/SPECT), cardiac magnetic resonance, or non-invasive assessment of myocardial function [32]. Extended follow up will be essential to monitor the durability of collateral formation and endothelial function, as well as to evaluate for possible adverse effects, such as maladaptive angiogenesis. By integrating a range of these mechanistic endpoints, future trials can more precisely define how these antidiabetic drugs influence coronary vascular biology and myocardial perfusion, ultimately guiding their use in IHD. SGLT2 inhibitors, GLP-1 receptor agonists, and DPP-4 inhibitors represent a new frontier in vascular therapeutics. Defining the clinical conditions for their application will be essential to realizing their full potential.

## Availability of Data and Materials

The data supporting the findings and conclusions are available in online publications and are available upon reasonable request. There are no confidentiality or sensitivity concerns regarding the data. Please contact the corresponding author for further details on data access.
